# Cell type-specific characterization of nuclear DNA contents within complex tissues and organs

**DOI:** 10.1186/1746-4811-1-7

**Published:** 2005-10-04

**Authors:** Changqing Zhang, Fang Cheng Gong, Georgina M Lambert, David W Galbraith

**Affiliations:** 1 Department of Plant Sciences, The University of Arizona, Tucson, Arizona, 85721, USA; 2 Operon Biotechnologies, Inc., 2705 Artie Street Bldg. 400, Ste. 27, Huntsville, AL 35805, USA

## Abstract

**Background:**

Eukaryotic organisms are defined by the presence of a nucleus, which encloses the chromosomal DNA, and is characterized by its DNA content (C-value). Complex eukaryotic organisms contain organs and tissues that comprise interspersions of different cell types, within which polysomaty, endoreduplication, and cell cycle arrest is frequently observed. Little is known about the distribution of C-values across different cell types within these organs and tissues.

**Results:**

We have developed, and describe here, a method to precisely define the C-value status within any specific cell type within complex organs and tissues of plants. We illustrate the application of this method to *Arabidopsis thaliana*, specifically focusing on the different cell types found within the root.

**Conclusion:**

The method accurately and conveniently charts C-value within specific cell types, and provides novel insight into developmental processes. The method is, in principle, applicable to any transformable organism, including mammals, within which cell type specificity of regulation of endoreduplication, of polysomaty, and of cell cycle arrest is suspected.

## Background

The amount of DNA contained within a haploid nucleus of eukaryotic organisms is termed the C (constant) value [1]. For many eukaryotes, the nuclei of somatic cells contain a 2C DNA amount, and the growing cells participate in a simple mitotic cell cycle in which four temporally-linked phases, G1, S, G2 and M, serve to separate the processes of DNA duplication (S-phase) from chromosomal segregation (M-phase). Monosomatic tissues containing mitotically active cells therefore are characterized by cells having nuclear DNA contents ranging from 2C to 4C, depending on the position of the cells within the cell cycle. The proportions of cells within these phases is a function of the proportions of cells that are actively cycling and the degree of cycle synchrony, and evidently reflects also whether or not the cells are arrested at particular points within the cell cycle, most commonly G0/G1 (2C) or G2 (4C). In polysomatic tissues, the situation is complicated by the occurrence of an alternative cell cycle, termed endoreduplication, in which successive S-phases are not followed by M-phases. This produces uninucleate cells having multiplicative DNA contents (2^n ^C, where n = 1,2,3..., for most sources of somatic cells, and 3 × 2^n-1 ^C for the endoreduplicated endosperm derived from triploid progenitor cells). Polysomaty is particularly common in higher plants [2]; for some species, such as *A. thaliana*, it is encountered throughout the mature tissues of the organism [3], while in others it is restricted to specific tissues [4].

The functional significance of the state of the nuclear C-value at which DNA synthesis arrests remains obscure, in part due to a lack of facile and precise methods for identifying its occurrence as a function of specific cell types. It is clear that, in the analysis of developmental gene expression and the cell biology underlying its regulation, the nuclear C-value represents an important parameter reflecting both the cell cycle status of the cell within which the nucleus is located, as well as the participation of the cells of polysomatic tissues within cycles of endoreduplication. Conversely, the regulated arrest of the cell at specific nuclear C-values reflects the activities of regulatory mechanisms about which we know very little.

We wondered if our flow cytometric methods for analysis of nuclear C-values [5] might be combined with transgenic expression of a nuclear-targeted version of the Green Fluorescent Protein (GFP) placed under the regulation of cell type-specific promoters, thereby permitting analysis of the C-value status of specific cell types. We reasoned that the labeling of nuclei of specific cell types with GFP would allow their detection using flow cytometry and, via simultaneous biparametric analysis of DNA content, lead to their assignment to various C-value classes. In this report, we validate this experimental approach, describing recombinant DNA constructions that encode Fluorescent Protein (FP)-fusions that are appropriately targeted to the nucleus, and which are quantitatively retained within the nuclei following cell homogenization. We go on to describe conditions for confocal examination of transgenic plants exhibiting a number of different cell-type specific patterns of expression, and for flow cytometric analysis of homogenates prepared from these plants. We finally employ the method to uncover evidence of cell type-specific arrest of particular cell types within different C-value states. The significance of these observations is discussed.

## Results

The proposed experimental concept requires that it be possible to target GFP, or other Fluorescent Proteins, to the nuclei of transgenic plants under the control of cell type-specific promoters, that the nuclei display sufficient fluorescent signal to be detectable by microscopy and flow cytometry, that the GFP-based signal not interfere with counterstaining and flow analysis of nuclear DNA content, and that the GFP-based fluorescence be retained within the nuclei following homogenization and during flow analysis. To be maximally useful, the concept and the procedures should be applicable to plants having small (cf. *A. thaliana*) as well as larger genomes.

**Figure 1:**
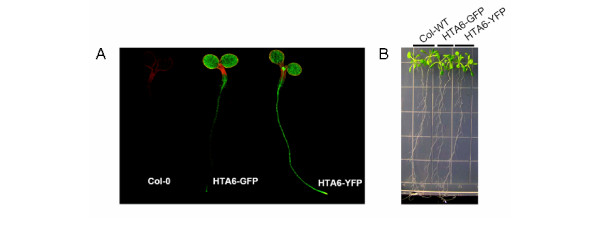
Confocal and bright-field images of wild-type plants, and plants transgenically expressing HTA6-GFP and HTA6-YFP under the transcriptional control of the CaMV35S promoter. For the bright-field picture, seeds of the three genotypes were germinated on MS agar plates. 3-day-old, similar-sized seedlings were transferred onto fresh MS agar plates, and were grown in a vertical position for two weeks.

**Figure 2:**
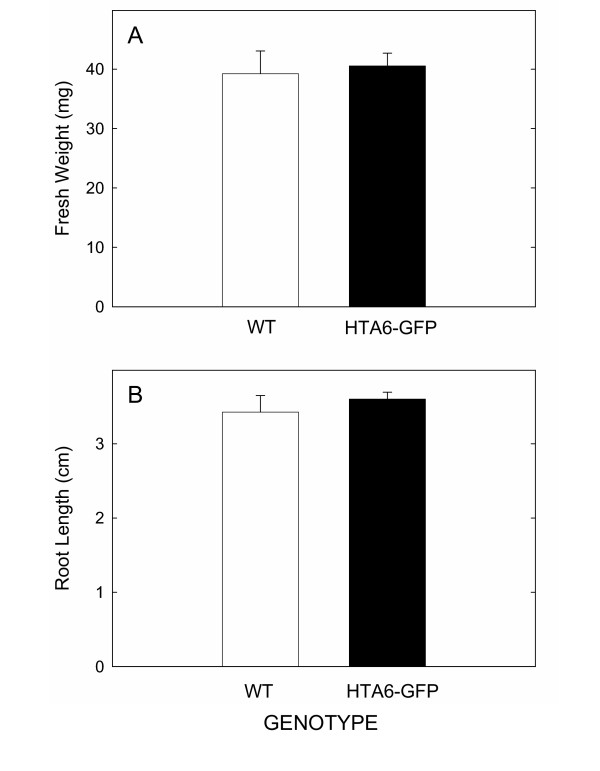
**(A) **Fresh weights and **(B) **root growth amounts of plants that are transgenic or non-transgenic for HTA6-GFP expression. Measurements were made on three groups of 20 pooled plants, and the error bars indicate standard deviations. Abbreviations: WT, wild-type; HTA6-GFP, transgenic plant expressing HTA6-GFP.

To test this concept, we employed *A. thaliana*, a model plant species for which a uniquely comprehensive amount of molecular information is available. *A. thaliana *also comprises one of the smallest nuclear genomes within the flowering plants [6], thereby providing an excellent test of the lower limit of resolution of the methods. For nuclear labeling, we evaluated the performance of a number of different translational fusions of nuclear proteins with GFP. Optimal for our purpose was a fusion of GFP with the coding region of a histone 2A gene (HTA6; At5g59870). Under the transcriptional control of the Cauliflower Mosaic Virus (CaMV) 35S promoter, transgenic *A. thaliana *plants expressing HTA6-GFP were phenotypically normal, and displayed brightly fluorescent nuclei within all parts of the plant (Figure 1). Nuclei of similar brightness were seen for transgenic plants expressing HTA6-YFP. No effects of transgenic GFP expression were detected on plant fresh weights or root growth rates (Figure 2), or by using whole genome long oligonucleotide microarrays to monitor alterations in gene expression (unpublished data).

**Figure 3:**
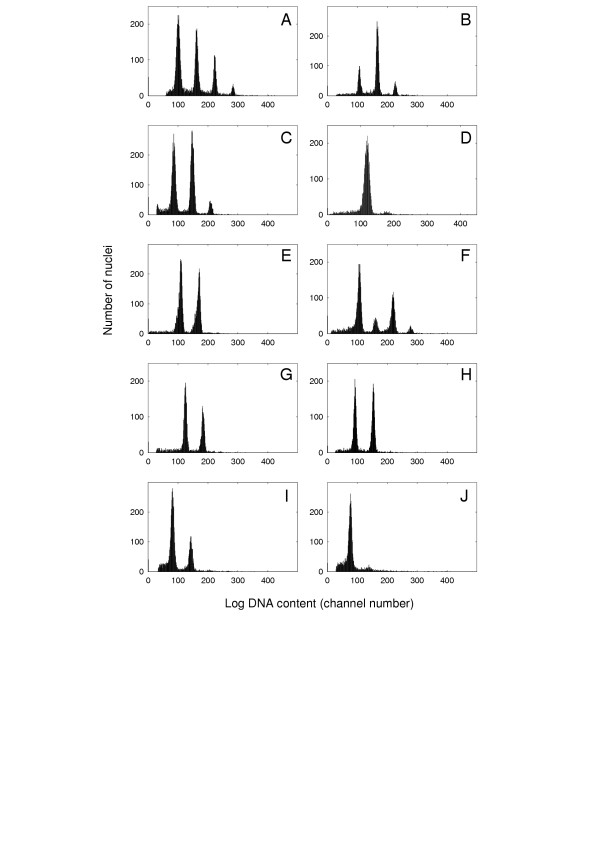
**Flow cytometric analysis of the nuclear DNA contents of plant root homogenates. **Homogenates, prepared as described by Galbraith *et al*. [5], stained with DAPI, were analyzed using a Partec CCAII Flow Cytometer (Partec GmbH, Munster, Germany). The instrument PMT and amplification settings were adjusted between samples to provide distributions conveniently distributed across the abscissae. This means that the numerical positions of the nuclear peaks should not be used for comparative analysis of DNA content between species. **(A) ***Arabidopsis thaliana*. **(B) ***Pisum sativum*. **(C) ***Cucumis sativus*. **(D) ***Daucus carota*. **(E) ***Nicotiana tabacum*. **(F) ***Lycopersicon esculentum*. **(G) ***Petunia hybrida*. **(H) ***Vinca rosea*. **(I) ***Zea mays*. **(J) ***Oryza sativa*.

We concomitantly chose to focus on plant roots: roots of many important crops and model species either are polysomatic or comprise a large proportion of cells arrested at a 4C nuclear DNA content (Figure 3). For example, uniparametric flow analysis of root homogenates prepared from the apical 1 cm regions of the primary root of ten day-old *A. thaliana *plants identifies four populations of nuclei (Figure 3A), equally spaced along the DNA content axis (logarithmic scale) corresponding to nuclei respectively having 2C, 4C, 8C, and 16C DNA contents. Polysomaty was also observed for root tips of cucumber, pea, and tomato. For the other species examined (tobacco, *Vinca*, maize, rice, and carrot), polysomaty was absent, but for maize, tobacco, petunia and *Vinca*, a large minority of the nuclear populations represented cells having 4C nuclei (see also [5]). Our observations are consistent with other compilations [4].

**Figure 4:**
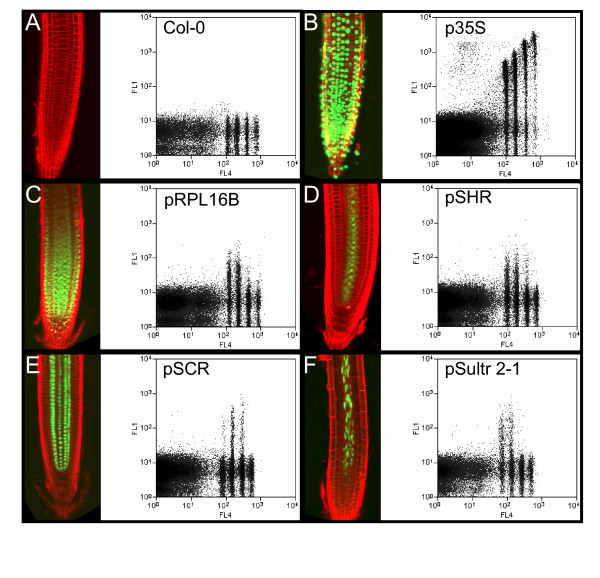
**Confocal and biparametric flow cytometric analyses of wild-type and transgenic plants expressing nuclear GFP. **Flow cytometry was done using a Cytomation MoFlo flow cytometer with laser excitation at 365/488 nm, and biparametric detection of DAPI fluorescence (418–482 nm; FL4; log units), and GFP fluorescence (505–530 nm; FL1; log units), with triggering based on 90°-light scatter [59]. For confocal microscopy, roots were counterstained by dipping in propidium iodide (1 μg/mL in water) for 2 minutes. Abbreviations: p35S: CaMV 35S promoter; pSCR: SCARECROW promoter; pSHR: SHORTROOT promoter; pRPL16B: ribosomal protein large subunit 16B promoter; pSultr2-1: sulfate transporter 2-1 promoter.

**Figure 5:**
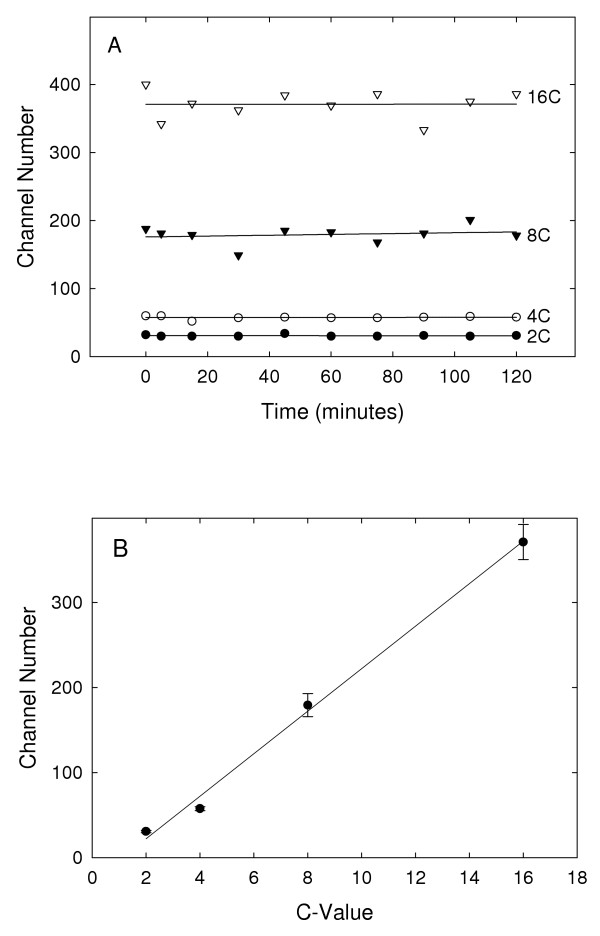
**Analysis of the stability and amounts of targeted GFP fluorescence within nuclei following homogenization. **Biparametric flow cytometric analyses were done of transgenic plants constitutively expressing nuclear GFP at various times following homogenization. **A. **The modes of the GFP fluorescence distributions for the four classes of nuclei (2C, 4C, 8C, 16C) are plotted as a function of time after homogenization. **B. **The modes obtained from the GFP fluorescence distributions were averaged across the time course for the different nuclear classes, and these mean values are plotted against C-value. Error bars indicate standard deviations.

**Figure 6:**
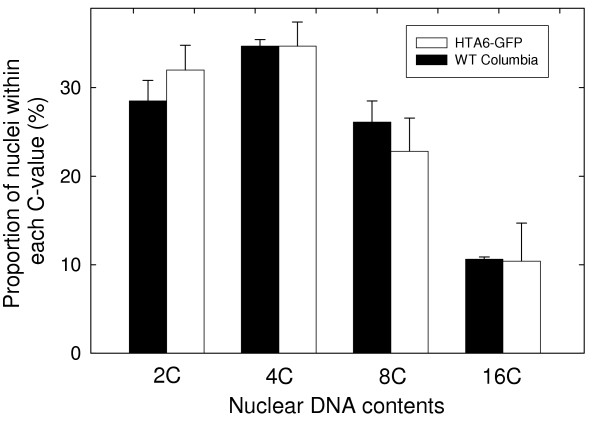
**Comparison of the distributions of nuclei within the various C-value classes found in the roots of wild-type plants and plants transgenic for expression of HTA6-GFP. **Measurements were made on three replicate samples (wild-type and transgenic) each sample comprising ~100 pooled seedlings. The error bars indicate standard deviations.

Examination of the roots of wild-type and transgenic *A. thaliana *plants expressing HTA6-GFP was done via confocal microscopy. The confocal images and the corresponding biparametric flow cytometric analyses of the GFP and DNA contents of their nuclei are presented in Figure 4. Wild-type plants display no nuclear GFP fluorescence and, in the flow analysis (Figure 4A), the four populations of nuclei, corresponding to the 2C, 4C, 8C, and 16C nuclei, are located close to the abscissa. In comparison, the roots of plants constitutively expressing HTA6-GFP under the control of the CaMV 35S promoter contained green-fluorescent nuclei, and the flow histograms display clusters of nuclei corresponding in DNA content to 2C, 4C, 8C, and 16C but also producing a GFP signal that increases with DNA content (Figure 4B). The intranuclear GFP fluorescence was stable over the period of time following homogenization required for the flow analyses (Figure 5A), and the amounts of intranuclear GFP fluorescence scaled linearly with nuclear DNA content (Figure 5B). Finally, the proportions of nuclei within the different C-value classes were not significantly different when wild-type and transgenic plants were compared (Figure 6). Within the apical 10 mm of the *A. thaliana *primary root, therefore, exist 2C, 4C, 8C, and 16C cells, and the nuclei of these cells appear equally capable of accumulating GFP-labelled histone H2A.

**Figure 7:**
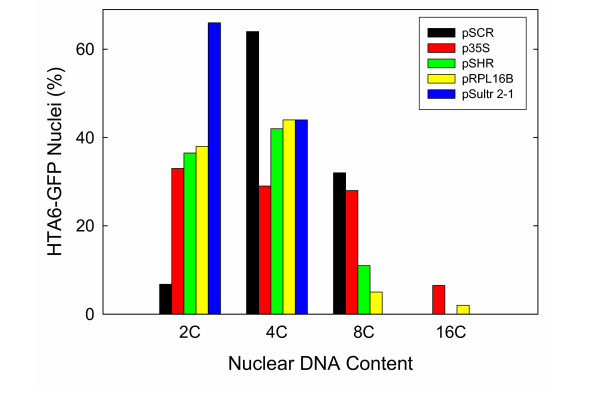
**Quantification of the proportions of nuclei within the various C-value classes for transgenic plants expressing HTA6-GFP under the control of constitutive and cell type-specific promoters. **The proportions were determined by integrating the total number of events within the biparametric plots of Figure 4 falling within a rectangular box containing the specific C-value class and having a GFP-intensity value of 18 or greater. Abbreviations: See Legend to Figure 4.

We next wondered whether the presence of nuclei of different C-value classes might be associated with specific cell types or root sub-regions. To address this question, we produced transgenic plants expressing HTA6-GFP under the control of both cell type-specific and region-specific regulatory sequences. Transgenic plants expressing HTA6-GFP under the control of the *Sultr*2-1 promoter [7] exhibited nuclear GFP fluorescence restricted to the phloem companion cells (PCC; Figure 4F). Regulation of HTA6-GFP expression by the promoters of the *SCARECROW *(*SCR*), and *SHORTROOT *(*SHR*) genes resulted in a restriction of GFP fluorescence (Figures 4D and 4E) respectively to nuclei of the endodermis, the cortex/endodermal initials, and the quiescent center, and to nuclei of the stele (the pericycle and internal vascular tissue) [8,9]. Regulation of expression by the promoter of a gene encoding protein 16B of the large ribosomal subunit resulted in nuclear fluorescence more generally localized to the meristematic region (Figure 4C). Flow cytometric analysis of homogenates indicated that PCC and the stele exclusively contained 2C and 4C nuclei, as did the cells within the meristem. In contrast, endodermal cells predominantly contained 4C and 8C nuclei (Figure 7).

**Figure 8:**
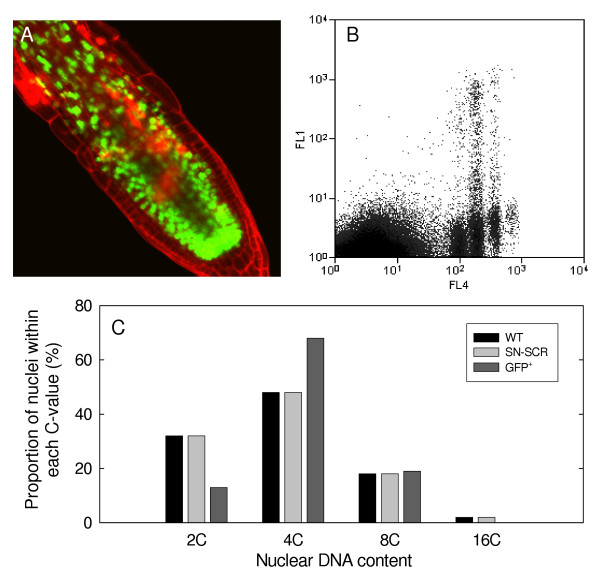
**Production of supernumerary endodermal cell layers is associated with accumulation of 4C and depletion of 2C cells. **Supernumerary endodermal cell layers were produced by transgenic expression of SHR under the control of the SCR promoter, and these layers were identified via expression of HTA6-GFP under the control of the SCR promoter. **A. **Confocal analysis of the transgenic plants. **B. **C-value distributions of GFP-positive and -negative nuclei as determined through biparametric flow analysis (axis designations as described in the Legend to Figure 4). **C. **Classification of the proportions of nuclei within the various C-value classes for non-transgenic controls (WT), for all nuclei within transgenic plants producing supernumerary endodermal cells (SN-SCR), and for GFP-positive nuclei (GFP^+^) within these same transgenic plants.

To explore whether the occurrence of 4C and 8C nuclei was directly correlated with SCR expression, we examined the distribution of C-values of GFP-positive nuclei within transgenic plants producing supernumerary endodermal cell layers, as a consequence of ectopic expression of SHR under the control of the SCR promoter [10]. These transgenic plants contained various proportions of such supernumerary cells, clearly identified by the presence of nuclear GFP (Figure 8A). The proportion of GFP-positive 4C nuclei was dramatically elevated as compared to the wild-type control and as compared to the total distribution of nuclei within the transgenic plants (Figure 8B, 8C). In contrast, no differences were seen in the proportions of all nuclei within the various C-value classes when transgenic and wild-type plants were compared.

## Discussion

The described method relies on the targeting of GFP to the nucleus, and its retention within the nucleus during cellular homogenization and flow cytometric analysis. In previous work, we described the use of a tobacco nuclear localization signal to target a chimeric protein comprising the complete coding region of β-glucuronidase fused to GFP [11,12]. Although such a molecule is effectively targeted to the nucleoplasm *in vivo*, it appears to slowly leak out of nuclei following homogenization. This is not an issue for the flow cytometric analysis of nuclei having large DNA contents, such as tobacco, since the nuclear GFP signal remains well above the background detection level of the flow cytometer for reasonable periods of time following homogenization [12]. In contrast, for plants having small nuclear genome sizes, such as *A. thaliana*, the small size of the nuclei and the low amplitude of the GFP-signal, coupled to leakage of the targeted molecules, means that nucleoplasmic targeting is unsuitable for flow cytometric analysis of isolated nuclei. This problem can be avoided by employing as the targeting signal a nuclear protein that represents a structural component of the nucleus, in this case histone HTA6. Constitutive transgenic expression of the HTA6-GFP fusion protein has no detectable effect upon plant growth or development. Interestingly, constitutive expression of the GFP-HTA6 (i.e. in a reversed orientation) fusion protein also had no perceptible effects on growth and development (data not shown). This is consistent with our understanding of the three-dimensional structures of histones [13]. For HTA6, both the N- and C-termini are exposed at the nucleosomal surface and, of the 13 predicted *A. thaliana *H2A proteins (HTA; ), HTA6 has the second longest N-terminus (14 aa), and the longest C-terminus (21 aa).

The patterns observed within the two-dimensional frequency distributions produced by flow cytometry indicate that a majority of the root cells of transgenic plants contain green fluorescent nuclei, which confirms that the CaMV 35S promoter is active during the development of the different cell types present within the region analyzed [14]. The fact that HTA6-GFP fluorescence scales linearly and very precisely with DNA content implies the accumulation of nuclear histone 2A is tightly correlated with DNA content. This observation, coupled to the lack of leakage of HTA6-GFP from the nuclei in homogenates, is consistent with the hypothesis that most of the HTA6-GFP is complexed within chromatin rather than being free within the nucleoplasm.

Although the two-dimensional frequency distributions provide unambiguous identification of the DNA content values of GFP-labelled nuclei, for these to be meaningful, it is crucial that expression of HTA6-GFP not perturb the system under study. As far as we can tell, this appears to be the case: no phenotypical differences were seen between transgenic and wild-type plants, nor were differences seen in the proportions of nuclei within the various C-value categories. It should be noted that the same flow cytometric strategy should be technically applicable to transgenic plants expressing any GFP (or other FP) fusion that is targeted to and retained within the nucleus, with the same caveat that such expression not perturb the system under study.

The observation of cell type-specific patterns of C-value suggests that increasing nuclear DNA content represents one strategy evolved by multicellular organisms to specify cell types. As far as we are aware, this is the most precise experimental evidence supporting this rather simple idea in higher plants, and application of this method to other situations in which increased DNA content is associated with cell type differentiation should rapidly provide a valuable body of data. For example, in other work, it has been hypothesized that basal cells within the xylem pericycle cells arrest in G2, thereby becoming susceptible to auxin-mediated signals that trigger the first formative divisions leading to lateral root initiation [15-18]. Consistent with this hypothesis, genes characteristic of the G2/M boundary are coordinately induced in *A. thaliana *shortly after imposition of conditions leading to synchronized induction of lateral roots [18].

In both of these situations, cell type specification appears associated with an increase in the proportion of cells containing 4C nuclei. At the cytological level of analysis, such a situation can arise through G2 arrest of cells within a monosomatic diploid cell cycle, or, equally-well, through G1 arrest of cells that have entered the first endoreduplicative cell cycle (i.e. having become tetraploid). Complicating cytological analysis is the potential for formation of polytene chromosomes. Further experiments will evidently be required to clarify the situation, and methods of *in situ *hybridization utilizing endogenous [19-22] and transgenic chromosomal markers [23] should prove invaluable.

At the molecular level, many candidates for cell cycle regulators responsible for accumulation of 4C cells can be identified from the existing body of knowledge for other eukaryotic organisms [24,25], and that emerging for plants, particularly *A. thaliana*. These include cyclins that are specifically active during the G2/M transition [26], mitotic cyclin-dependent kinase and its activators and inhibitors [27-34], the anaphase promoting complex (APC) and components that interact with the APC [24,35,36], and molecules associated with check-points relating to cell size [37,38], radiation damage [36,39], and spindle assembly [36].

The mechanisms regulating cell cycle status and nuclear DNA content within the endodermal and cortical cells may also reflect the nature of the *SCR *and *SHR *genes, which encode members of the GRAS-STAT family of transcription factors [40,41]. Other members of this family have been shown to interact with regulators of the cell cycle [42-44]. One role of SHR and/or SCR may be to arrest the diploid cell cycle within endodermal cells at the G2/M boundary, and perhaps also to regulate an endoreduplicative event converting these nuclei from a G2 to a G1 state without an intervening M-phase. Of the 30 genes found to be most strongly up- or down-regulated within the endodermis [45], one candidate for a regulatory role is At5g26900, since it exhibits homology to *fizzy1 *of *X. laevis *which is required for APC activation in that system [46].

Interestingly, recent reports implicate expression of additional members of the GRAS-STAT family in the establishment of nodulation in *Medicago truncatula *[47,48]. We note that the flow cytometric method described here should be appropriate for unambiguous determination of the C-value status of root initials responsive to nodulation signals, and of the different cell types that subsequently develop during root nodule formation. If nodule development can be shown to specifically involve G2-arrested cells within the root cortex, this would imply co-option of cellular mechanisms that normally regulate lateral root formation.

In general, the method outlined in this report should be applicable to any transformable plant species within which the regulated expression of HTA6-GFP results in fluorescent nuclei. For promoters of low activity, coupling of nuclear GFP expression to amplification systems (for example provided by GAL4/VP16 [49]) may be required. Orthologues of histones 2A should be readily accessible for most species, and we have established that a GFP fusion to the rice HTA6 orthologue is targeted to the nucleus in transgenic rice plants (CQZ, C. Santhosh Kumar, V. Sundaresan, and DWG, unpublished results). Plant cell types for which a determination of nuclear DNA content should be of particular interest include those undergoing regulated endoreduplicative cycles, such as found within developing seed storage tissues [50], within developing trichomes [51], and in the establishment of symbioses [52], since the method is not restricted to cells operating within a conventional diploid cell cycle. The method should also be helpful in clarifying reports of the unexpected onset of reductive mitoses within endoreplicated cells [27]. It should also be possible to develop multiparametric flow cytometric methods combining the identification of the C-values of nuclei of specific cell types with a determination of the occurrence of S-phase (relying on antibody-based detection of bromodeoxyuridine incorporation [53]). This would allow direct determination of the onset of DNA replication particularly within endoreduplicating cells at various C-value levels, thereby providing a greatly increased degree of sophistication in the analysis of processes of this type. The method should also be applicable to lower plants, and could be readily tested using *Physcomitrella*, which can be transformed and for which the specific G2-arrest within the chloronema has been recently described [54].

Finally, it should also be noted the flow cytometric method should be equally applicable to transformable non-plant species, including mammals. The relevance of endoreplication to mammalian development, both under normal and abnormal circumstances, is increasingly evident [55,56], and the ability to accurately chart its occurrence within specific cell types should prove important in the analysis of development as well as of specific disease states.

## Methods

### Recombinant constructions

All general molecular manipulations were done according to standard procedures [57]. *PfuUltra*™ high-fidelity DNA polymerase (Stratagene, La Jolla, CA, USA) was used for PCR-based amplification of fragments for cloning.

To construct a T-DNA binary vector for expressing GFP in plants, a 2445 bp fragment covering the sGFP expression cassette was released with HincII and SspI from pGFP-JS (Jen Sheen, Massachusetts General Hospital, Boston MA), and inserted into pCAMBIA1302 between the SmaI (9755) and PmlI (752) sites. For convenience of discussion, we call this reassembled vector pCsGFPB. The *A. thaliana *core histone HTA6 coding sequence (450 bp) was PCR amplified from a cDNA first strand preparation, using forward primer 5'- CATGCCATGGAATCCACCGGAAAAGTG-3' and reverse primer 5'- CATGCCATGGCAGCTTTCTTTGGAGACTTGACTG-3'. The cDNA first strand was prepared using reverse transcriptase SuperScript II according to the manufacturer's recommendations (Invitrogen, Carlsbad, CA, USA). The amplified fragment was inserted into the NcoI site of pCsGFPB. This resulted in in-frame fusion of HTA6 to the N-terminus of GFP, which is downstream of an enhanced CaMV 35S promoter. In the coding region of HTA6, a single nucleotide change at position 118 (G to A) was confirmed by sequencing analysis. This single nucleotide change leads to a point mutation (I39V), and this mutation is retained in all derivative constructs.

Vector pCsGFPB carrying the HTA6 coding sequence was further modified by removing the stop codon (TAA) of the GFP open reading frame as well as the following 14 bp. This modification shifts the contiguous BamHI and XbaI sites to the sGFP open reading frame, and leads to three amino acids (Gly-Ile-Leu) being added to the original sGFP, with the "TAG" within the XbaI site becoming the new stop codon. Then a 70 bp computer generated random sequence (CGAATGTAGTACGTATTCTCCGAACTGAAGCACCTGAGACGTGTAATGTCGGGCCATCTCATACGTACGG) was inserted immediately after the new stop codon, to serve as a transcriptional tag for monitoring the sGFP mRNA level using microarrays printed with the appropriate complementary sequence. Finally, the CaMV 35S promoter (780 bp) upstream of sGFP was excised using EcoRI; and an attR cassette (Frame C, Invitrogen, Carlsbad, CA, USA) was installed. This Gateway-adapted vector was named pCGTAG, and it was used in making the remaining constructs in this study.

Genomic sequences immediately upstream of the start codons of the SCARECROW (SCR), Sulphate Transporter 2-1 (Sultr2-1), SHORTROOT (SHR), and RPL16B coding sequences were PCR amplified. A 2131 bp fragment for SCR was amplified with forward primer 5' CACCGGATAAGGGATAGAGGAAGAGG 3' and reverse primer 5' GGAGATTGAAGGGTTGTTGGTCG 3'. A 2048 bp fragment for Sultr2-1 was amplified with forward primer 5' CACCGCTGACAACTAACACTCCTC-3' and reverse primer 5' CTTCTTCTCGAGTTTTGACGTTGTG 3'. A 2495 bp fragment for SHR was amplified with forward primer 5' CACCGGACAAAGAAGCAGAGCGTGG 3' and reverse primer 5' TTAATGAATAAGAAAATGAATAGAAGAAAGGGAGACCCAC 3'. A 1074 bp fragment for RPL16B was amplified with forward primer 5' CACCTTTCCCACCTCTCTTCAACTTC 3' and reverse primer 5' CGTAAATAGTAAGTTAAATCCCCAAAACGAGGAACG 3'. The five fragments were then cloned into the Gateway entry vector pENTR/D-TOPO (Invitrogen, Carlsbad, CA, USA). The plasmids carrying the regulatory sequences of SCR, Sultr2-1, SHR, and RPL16B were linearized with restriction enzymes EcoRV, EcoRV, EcoNI, DrdI, and EcoRV respectively. These linearized plasmids were employed in the Gateway LR reaction, to insert the individual regulatory sequences into the Gateway-adapted T-DNA binary vector pCGTAG.

### Plant transformation

Plasmids carrying the above constructs were introduced into *Agrobacterium tumefaciens *strain GV3101. *A. thaliana *'Columbia' (Col-0) was transformed using the floral dip method [58]. Seeds (T1) were harvested and selected on MS agar plates supplemented with 40 mg L^-1 ^hygromycin and 75 mg L^-1 ^carbenicillin. Roots from hygromycin-resistant seedlings were examined for GFP fluorescence using confocal microscopy. Confirmed transformants were transferred to soil.

### Introduction of pSCR-HTA6-GFP into plants carrying supernumerary endodermal cell layers

Transgenic *A. thaliana *seeds carrying SCRpro::SHR were kindly provided by Philip Benfey (Department of Biology, Duke University). The roots of this transgenic line have an increased number of cell layers which display characteristics of the endodermis [10]. We crossed this transgenic line with transgenic plants carrying pSCR-HTA6-GFP. F1 seeds were germinated on MS plates lacking antibiotics. Roots of three-week old seedlings were subjected to confocal and biparametric flow analyses.

### Confocal microscopy

Roots from T1 or T2 seedlings were counterstained with 1 μg ml^-1 ^propidium iodide, PI (Sigma, St Louis, MO, USA) for 1 to 2 minutes, and were placed on slides carrying a drop of water for observation. GFP fluorescence was imaged by confocal microscopy using a MRC 1024 MP (Bio-Rad, Hercules CA) confocal scanner attached to an Olympus BX-50 upright microscope, equipped with UPlanFl 4X/0.13, UPlanFl 10X/0.30, and UPlanApo 20X/0.70 objective lenses. LaserSharp2000 (Bio-Rad) was employed for image acquisition and Photoshop 5.0 (Adobe Systems Inc., San Jose, CA) for image processing.

### Growth comparisons

Seeds of Col-0 and of a homozygous transgenic line carrying CaMV35S-HTA6-GFP were sterilized and planted on MS agar plates (2% sucrose, 1.2% agar). The plates were kept at 4°C for three days before transfer to a Conviron growth chamber under 16 hour days / 8 hour nights, with an incident light flux of 150-175 μmol m-2 sec-1, and temperatures of 22°C (day) and 20°C (night). The plates were placed vertically for 3 days. Seedlings of similar sizes were then transferred onto fresh plates, each plate containing ten wild-type and ten transgenic seedlings. The position of the root tip of each seedling was marked on the bottom lid of the plate using a marker pen. Root elongation was measured five days after transfer, and the seedlings were then collected for measurement of fresh weights.

### Flow cytometry

Flow cytometry was done using a MoFlo flow cytometer (Dako Cytomation, Fort Collins, CO) equipped with a Coherent Enterprise II laser providing separate beam paths comprising 50 mW at 365 nm and 200 mW at 488 nm. Homogenates of *A. thaliana *roots were prepared by chopping [5], and nuclei were stained by addition of 2 μg/mL 4',6-diamidino-2-phenylindole (DAPI). Samples were analyzed at an event rate of 200 nuclei/s. GFP fluorescence, excited by the 488 nm beam, was detected following reflection by a 555DCLP dichroic beam splitter through a 530/40 band pass filter. DAPI fluorescence, excited by the 365 nm beam, was routed directly through a 450/65 band pass filter. Biparametric histograms of log DAPI versus log GFP signals were triggered on side scatter and collected to a total count of at least 100,000 events.

### Competing interests

The author(s) declare that they have no competing interests.

## Authors' contributions

CZ produced the constructions and the transgenic plants, characterized the transgenic plants using confocal microscopy, participated in the flow cytometric experiments, and contributed to the preparation of the manuscript. GL assisted in the confocal microscopy, performed the flow cytometric measurements, and contributed to the preparation of the manuscript. FG participated in the flow cytometric measurements, and contributed to the preparation of the manuscript. DG conceived of the study, participated in its design and coordination, and drafted the manuscript. All authors read and approved the final manuscript.
